# Investigating the role of HSP90 in cancer cell phenotypic plasticity

**DOI:** 10.46439/breastcancer.4.021

**Published:** 2024

**Authors:** Vincent Sollars, Alexandria Chapman, Nicole R. Liang, Seth Myers

**Affiliations:** 1 Department of Biomedical Sciences at Joan C. Edwards School of Medicine at Marshall University, USA

**Keywords:** Epidgenetic, Cancer, HSP90, Histone Acetylation, Phenotypic Plasticity, Multi-drug Resistance, Cellular Stress

## Abstract

*“What are the mechanisms driving tumor evolution under the selective pressure of chemotherapeutics?”* The emerging importance of epigenetic gene regulation in cancer progression necessitates not only our understanding of which genes are potential targets but also what mechanisms are employed in targeting those genes. Understanding the mechanisms that promote the evolution of the normal genome and epigenome is central to understanding how cancer cells adapt to chemotherapy. Our previous investigations have shown that heat shock protein 90 (HSP90) has a critical role in epigenetic gene regulation through histone acetylation and phenotypic plasticity. We recently extended these results in an A549 lung cancer model to test the role of HSP90 in the plasticity of cells regarding multi-drug resistance and epithelial-to-mesenchymal transition phenotypes. HSP90 is over-expressed in multiple cancers with poor prognosis. We propose that inhibition of HSP90 results in lower phenotypic plasticity of cancer cells making them more susceptible to chemotherapeutic intervention. Here we review the context of our results in the broader field of evolution of these phenotypes.

## Introduction

Life finds a way to survive, even malignant life. Viewing cancer as a Darwinian struggle between the body’s defense mechanisms and a rogue cell is an apt description of the process of oncogenesis. It also allows us to apply evolutionary biology principles to understand and design treatment strategies for those suffering from this malady. In particular, lung cancer with its high recurrence rate and early metastasis continues to plague therapeutic efforts [1]. Both metastasis and recurrence processes depend upon phenotypic change of cancer cells to adapt to new environmental conditions. The building blocks of evolution in these contexts is the genetic and epigenetic changes that different groups of cells can acquire after the initiation events of premalignancy. Recently, we have provided evidence showing that transient inhibition of Heat Shock Protein 90 (HSP90) by AUY-922 can inhibit epithelial-to-mesenchymal transition (EMT) associated with invasion and metastasis, as well as the up-regulation of drug transporters to a multi-drug resistant (MDR) phenotype associated with the emergence of chemotherapy-resistant cells [2]. In this review, we will show how this evidence supports the theory in the field.

## HSP90 and EMT

EMT is an important biological process involved with embryonic development and tissue regeneration. Epithelial cells will gain migratory and invasion abilities while losing cell polarity and cell-cell adhesion molecules [3]. This process is normally highly regulated. However, cancer cells will utilize EMT to take on more mesenchymal phenotypes. They become stem cell-like, able to dedifferentiate and self-renew. They lose E-cadherin, a cell adhesion molecule and powerful tumor suppressor. Thus, EMT allows cancer cells to proliferate rapidly and invade other tissues. EMT is a contributing etiology in many types of cancer, including breast, lung, prostate, and liver cancer. Increased EMT in tumors is associated with greater metastatic capacity, therapy resistance, and poor prognosis. The switch between epithelial and mesenchymal states is deregulated in cancer, and often, tumor cells remain in a hybrid epithelial-mesenchymal state [4]. Interestingly, breast tumors expressing both E-cadherin (an epithelial marker) and vimentin (a mesenchymal marker) yielded the worst survival rates. These transient changes contribute to a cancer cell’s phenotypic plasticity; the cell can cycle through various differentiation phenotypes based on environmental conditions [5]. In our experiments with A549 cells, we showed that cellular plasticity associated with the acquisition of EMT was inhibited by transient inhibition of HSP90 [2].

The process of EMT depends on genetic and epigenetic factors. EMT is initiated by signals from molecules such as Transforming Growth Factor-β (TGF-β), Wingless Integrin (WNT), and Phosphoinositide 3-kinase (PI3)/Protein kinase B (AKT). In our original experiments with HSP90 inhibition, we found that HSP90 inhibition triggered dysregulated expression of WNT in *Drosophila* [6]. The molecules activate pathways that govern the activity of several transcription factors (TFs), such as Snail 1/2, Twist, and Zinc finger E-box-binding homeobox (Zeb) 1/2. TGF-β and WNT directly regulate TF expression through Smad 2/3 (small” worm phenotype and Mothers Against Decapentaplegic protein family) and β-catenin interactions respectively. PI3/Akt can promote metastasis and chemoresistance through Twist phosphorylation. There is extensive crosstalk between the TFs to repress genes responsible for epithelial phenotypes while activating genes responsible for mesenchymal phenotypes. Among their functions, Snail 1 is partially responsible for the MDR phenotype through P-gp activation, Zeb 1 directly inhibits E-cadherin, and Twist up-regulates N-cadherin. These TFs are also under microRNA control. The binding of certain miRNA (mi-R) molecules to TF genes represses TF activity. Down-regulation of these miRs can result in upregulation of TF expression. Other microRNAs, such as mi-R155 and mi-R24, can act farther down EMT pathways to disrupt tight junctions and adherins. The aforementioned molecules are under TGF-β control as well [7]. Chromatin effectors also play a role in EMT induction. Snail 1/2 and Zeb 1 can recruit histone deacetylases (HDACs) and histone methyltransferases to epithelial genes to decrease their expression. One histone methyltransferase recruited is the Enhancer of Zeste Homeobox 2 (EZH2) complex, which represses the E-cadherin gene through inhibitory modification H3K27me3 at its promoter. Snail activity is also partially under epigenetic regulation, with Lysine demethylase 6B inducing its expression by removing repressive modifications from the Snail gene. EMT can also be induced by DNA methyltransferase 1 (DNMT1) and EZH2 as an early precursor to the activation of certain oncogenes like KRAS [8].

HSP90 contributes to cancer development through the promotion of EMT. HSP90 is up-regulated in malignant cells, and greater HSP90 levels in tumor cells correlate to lower survival rates [9]. HSP90 can epigenetically regulate EMT modulators to increase the incidence of EMT. Secreted HSP90 can signal the upregulation of EZH2 through the Extracellular Signal-regulated Kinase (ERK) pathway. The resulting signaling cascade suppresses E-cadherin, promotes EMT, and improves cancer cell migration. HSP90 can also activate TGF-β signaling through its client proteins Matrix Metalloproteinase-2 (MMP-2) and MMP-9 [10]. Interactions between HSP90 and Low-density Lipoprotein Receptor-related Protein 1 (LRP1) in various pathways can also trigger EMT. In colorectal cancer cells, HSP90 with LRP1 triggers the expression of Transcription factor 12 (TCF12), increasing fibronectin and decreasing E-cadherin [11]. In breast cancer cells, HSP90 and LRP1 work synergistically with clusterin to activate Akt, ERK, and Nuclear Factor Kappa-light-chain-enhancer of Activated B Cells (NF-κB), leading to EMT and increased metastatic capacity. HSP90 can also increase the expression of Snail 1/2, Twist, and Zeb ½ [12]. Multiple *in vitro* studies have shown that HSP90 inhibitors can suppress EMT, diminishing cancer cell growth and metastatic potential. Thus, there is great interest in utilizing HSP90 inhibitors as oncological therapeutics to combat EMT in tumors [13–15].

## MDR and Epigenetic Gene Regulation

MDR is understood as the nonreceptivity of a single cancer cell line to an assortment of anti-cancer drugs that vary in structure and function. MDR phenotypes within cancer cells can be demarcated into two categories: primary resistance and acquired resistance phenotypes. Primary resistance is associated with MDR abilities inherently found in a cell line before exposure to anti-cancer treatments, while acquired resistance transpires after exposure [16]. Cell expression of MDR can function by a variety of different processes, including elevated drug metabolism, heightened cellular efflux, growth factors, the increased ability to repair damaged DNA as well as a variety of other genetic factors [17]. It is these factors that have led to the attribution of over 90% of deaths in patients receiving chemotherapy and other anti-cancer drugs to MDR. In our experiments with A549 cells, we showed that cellular plasticity associated with the acquisition of MDR by heightened cellular efflux was inhibited by transient inhibition of HSP90 [2].

Of the different mechanisms of MDR, cellular efflux is of great interest. Efflux is defined as the ability of cells to transport hazardous chemicals out through transporters within the cellular membrane. An example of transporters associated with MDR include ATP-binding cassette (ABC) proteins, which include the Breast Cancer Resistance Protein (BCRP) and P-glycoprotein (P-gp) which is coded for on the ABC subfamily B member 1 gene (ABCB1) [17]. ABC transporters are membrane-spanning proteins participating in cholesterol homeostasis, transport of molecules in and out of cells and organelles, immune recognition, oxidative stress, and drug efflux. ABC transporters are considered the main arbitrators of multidrug resistance [18]. P-gp is overexpressed in about 50% of human cancers and this overexpression can be exacerbated by certain chemotherapies such as paclitaxel [17]. In our experiments, we found that ABCB1 and ABCC1 were up-regulated in response to paclitaxel and that this process was inhibited by prior transient inhibition of HSP90 [2].

One of the suspected causes of MDR is epigenetic alterations. Mechanisms such as hyper- and hypomethylation are associated with increased cellular access to oncogenes and decreased access to suppressor genes allowing for the continual proliferation of invasive cancer cells [16,19]. Previous work has shown that demethylation of the ABCB1 gene leads to a decrease in the amount of chemotherapy that is able to accrue within the cell [17]. Additionally, histone deacetylase inhibitors (HDACi) have been shown to modify the composition of transporters on the surface of some cancerous and noncancerous cells. HDACi have demonstrated the ability to increase acetylation and transcription factors within certain cells, which in turn have up-regulated of tumor suppressors and up-regulation of cell transporter expression, indicating that histone acetylation plays a role in ABC transporter regulation [18]. Our previous experiments were the first to link HSP90 to epigenetic dysregulation and histone acetylation in *Drosophila* which we later showed to be present in mammalian systems [6,20].

HSP90 is a cellular chaperone protein associated with epigenetic modification, and phenotypic change, and is often up-regulated within cancer cells. HSP90 is known for its ability to epistatically subdue or enable the expression of different genetic variants [21]. The level of the protein in cancer cells is associated with patient outcomes. High levels of the protein are correlated with high malignancy and less drug response [22]. In a study examining HSP90 in A2780 ovarian-originated cancer and its paclitaxel and cisplatin-resistant sublines, it was found that inhibiting HSP90 increased the susceptibility of the drug resistance cancers to paclitaxel and cisplatin. It was also found that silencing HSP90 decreased the level of P-gp and BCRP within the A2780 cells, suggesting that ABC transports are associated with or involved in HSP90-induced resistance to paclitaxel in ovarian cancer cells [23].

## HSP90 and Histone Acetylation

HSP90 and other heat shock proteins are overexpressed in a range of cancer cells [24]. We were the first to connect HSP90 function to epigenetic mechanisms, specifically to histone acetylation [6]. The expression of genes is partially regulated by histone modifications (e.g. acetylation) as a part of chromatin remodeling. Oncogenes or tumor-suppressor genes expression can be modified by changes in histone acetylation [25]. Tumor cells often lose p53 function and have higher expression of proto-oncogenes such as Human Epidermal Growth Factor Receptor 2 (HER2) and c-Myc [24]. Hsp90 is a molecular chaperone that involves itself with the post-translational processing, folding, stabilization, and maturation of various client proteins, including histone-modifying proteins. Several HDACs, which are enzymes responsible for removing acetyl groups from histones [26], and histone acetyltransferases (HATs), which are enzymes that add acetyl groups to histone proteins, are clients of HSP90. Since these are clients of HSP90, the regulation of HSP90 alters the balance of histone acetylation in cells. A multitude of histone modifications have been shown to be affected when HSP90 is inhibited in bladder cancer cells [27].

Cancer is highly adaptive, with a heterogeneous population that quickly responds to stress. This adaptability is crucial for cancer cells to survive and thrive in diverse environmental conditions, including those that would normally induce cellular stress and apoptosis. The heat stress response of cancer has been extensively studied, and it is becoming increasingly clear that cancer cells hijack normal cellular protective processes to survive [28,29]. A common feature of any cellular stress response is the effort to recover from protein misfolding through increased expression of molecular chaperones like HSP90. These responses to stress alter many cellular processes involved in malignancy (like the cases of EMT) [30].

Furthermore, the interplay between HSP90 and histone acetylation extends beyond individual gene regulation. Our results provide evidence that it contributes to the broader phenotypic plasticity seen in cancer cells, allowing them to switch between different states in response to varying environmental cues. As a key chaperone, heightened levels of HSP90 allow for the excessive accumulation and function of its client proteins [31]. It is common for proteins to require assistance to form their functional three-dimensional conformation when they are translated, but a subset of proteins have a significant amount of conformational instability in their mature state and require perpetual and ongoing chaperone assistance to avoid denaturation. Many of these “less stable” proteins are expressed in various cancer cells, and the higher levels of HSP90 expressed in cancer cells allow for their continued function. In addition, mutations that alter the three-dimensional structure of proteins, make malignant cells more reliant upon chaperone functions creating an addiction to high-level expression of HSP90.

## Phenotypic Plasticity and HSP90

**Figure 1.**
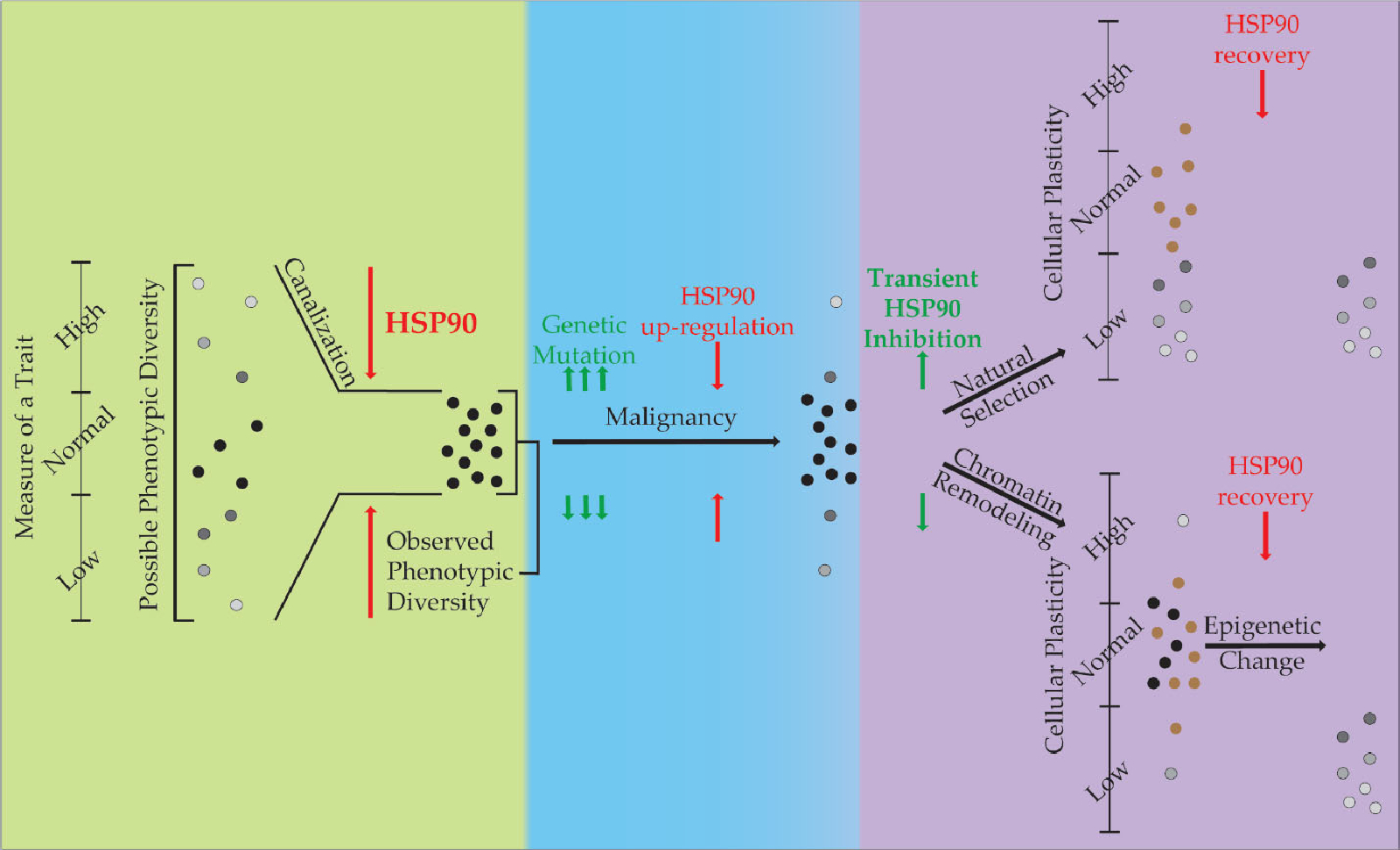
The Role of HSP90 in Phenotypic Diversity and Plasticity. This diagram describes the role HSP90 performs in canalization of phenotypes during evolution (green), its role in restriction of phenotypes during malignancy (blue), and two hypotheses for its role in our experiments with regards to phenotypic plasticity (purple). Phenotypic variance is displayed vertically with HSP90 restricting its expression through canalization. Cells that exist within the ‘normal’ phenotypic range are in black, while others are shown in grayscale. Cells shown in brown are those that die during inhibition by HSP90.

The first researchers to connect HSP90 to evolutionary biology did so in a variety of organisms including *Arabidopsis* and *Drosophila* [32,33]. We later showed that these mechanisms, at least partially, occur through epigenetic mechanisms involving histone acetylation [6]. It was later shown that HSP90 could inhibit the evolution of resistance to hormonal therapies in breast cancer [34]. This was an exciting discovery as it suggested that evolutionary principles such as canalization, which have been associated with HSP90 since Suzanne Rutherford and Susan Linquist linked HSP90 with evolution, could be applied to cancer biology. It is interesting to note that Conrad Waddington, the researcher who first coined the term epigenetics, also was the first to posit the process of canalization. The predominant success of the complex process of development despite the necessity of high precision in cell fate decisions and spatial relationships suggests a mechanism for buffering for minor changes. Canalization theory states that this is the result of canalization of the preferred normal phenotype of the species during development (Figure 1). The expression patterns of genes from among organisms in a particular species are diverse enough to give a wide range of phenotypes that are not present in the population (depicted on the left with a y-axis of phenotypic range). The present morphology of the species is favored and variations in gene expression that would result in deviation from this model are compensated for by canalizers (or capacitors) present in the system. In this context, HSP90 stores genetic and epigenetic variation, until stress releases that change allowing for rapid adaptation to the environment. In effect, HSP90 restricts the production of phenotypic diversity (canalization) to the normal distribution of phenotypes under non-stressful conditions.

The attribute of phenotypic plasticity, where an organism or cell can change its characteristics is antithetical to canalization that restricts variability in phenotype. If you apply the principles of canalization to oncogenesis as has been commonly done with the Darwinian evolution paradigm (Figure 1), HS90 becomes an important factor in the molecular evolution of malignant cells. When canalization theory is applied to malignancy, where genetic mutation and the epigenetic changes are acquired by cells to increase variability, the associated stress can release canalization and promote the acquisition of the Hallmarks of Cancer [35,36]. However, because mutation is a stochastic process, in which deleterious mutations accumulate and hinder the cell’s viability. To compensate for the protein folding problems associated with these mutations, malignant cells commonly up-regulate HSP90 expression 2–10 fold. Through this process, these cells acquire an addiction to HSP90 and an increased canalization capacity, though this capacity is often occupied in these cells (the ratio of complexed: uncomplexed HSP90 is much higher in malignant cells compared to normal cells) [37].

Our experiments were designed to test the effects of inhibition of HSP90 on phenotypic plasticity associated with the acquisition of the MDR and EMT phenotypes. These phenotypes are a form of cellular adaptation through plasticity. Two hypotheses that explain our results are presented (Figure 1). While understanding that these are not mutually exclusive, there is support for both. If natural selection is causing the change in cellular plasticity, then selection after transient treatment with an HSP90 inhibitor results in a pool of surviving cells that have less cellular plasticity. This could be due to the Darwinian selection of cells that have a higher free HSP90 level (and thus a higher canalization ability) and are thus less susceptible to inhibition. When HSP90 levels are restored, the increased canalization ability of the malignant cells exerts itself and restricts phenotypic plasticity when the cells are induced to undergo EMT or MDR. Alternatively, epigenetic changes also associated with HSP90 inhibition may result in a pool of surviving cells with less phenotypic plasticity. A multitude of histone modifications have been shown to be affected when HSP90 is inhibited in bladder cancer cells [27]. In addition, we found that the application of HDACi was able to reverse HSP90-mediated phenotypic effects in *Drosophila* and murine stem cells [6,20].

## Conclusions

If loss of phenotypic plasticity through HSP90 inhibition prevents the emergence of chemotherapy resistance, then a new mechanism for treating cancers will have been discovered. Our data show that some epigenetic impacts of HSP90 inhibition are conserved in mammalian systems and offer a mechanism for the loss of phenotypic plasticity. Since epigenetic changes occur in most forms of cancer and phenotypic plasticity is reliant upon them, further elucidation of the connections between epigenetic gene regulation, HSP90, and phenotypic plasticity could have clinical implications on treating advanced cancers. This would provide new treatment strategies in clinical studies using HSP90 inhibitors, particularly as adjuvant therapies to prevent the evolution of therapy resistance.

Eighty-five percent of lung cancers are non-small cell lung cancers (NSCLC) [38], with metastasis and ABC transporter-driven chemotherapeutic resistance as common features of these cancers either at the time of diagnosis or manifesting during the treatment process [39–44]. The development of drug resistance is a frequent problem in the management of breast cancer [45]. Many patients’ tumours are resistant to the therapeutic agents by the time of disease recurrence, leading to the average five-year survival rate of patients with metastatic breast cancer of 31% [46]. The recent evidence that some treatment approaches may incidentally enable the metastatic cascade [47–49] and the persistence of drug-resistant relapse after current treatment methods [50] demand new, rational, treatment strategies that can curtail the emergence of metastatic and drug-resistant cancer phenotypes simultaneously. Therefore, discovering ways to limit metastasis and chemotherapeutic resistance in cancer will benefit many patients.

## References

[1] Dholaria B, Hammond W, Shreders A, Lou Y. Emerging therapeutic agents for lung cancer. Journal of Hematology & Oncology. 2016 Dec;9:138. 27938382 10.1186/s13045-016-0365-z PMC5148871

[2] Bacon NA, Larre I, Lawag AA, Merritt II C, Smith M, Rosolen M, Low dose HSP90 inhibition with AUY922 blunts rapid evolution of metastatic and drug resistant phenotypes induced by TGF-β and paclitaxel in A549 cells. Biomedicine & Pharmacotherapy. 2020 Sep 1;129:110434. 32768937 10.1016/j.biopha.2020.110434 PMC7484166

[3] Ribatti D, Tamma R, Annese T. Epithelial-mesenchymal transition in cancer: a historical overview. Translational Oncology. 2020 Jun 1;13(6):100773. 32334405 10.1016/j.tranon.2020.100773 PMC7182759

[4] Davies A, Zoubeidi A, Beltran H, Selth LA. The transcriptional and epigenetic landscape of cancer cell lineage plasticity. Cancer Discovery. 2023 Aug 4;13(8):1771–88. 37470668 10.1158/2159-8290.CD-23-0225 PMC10527883

[5] Gupta PB, Pastushenko I, Skibinski A, Blanpain C, Kuperwasser C. Phenotypic plasticity: driver of cancer initiation, progression, and therapy resistance. Cell Stem Cell. 2019 Jan 3;24(1):65–78. 30554963 10.1016/j.stem.2018.11.011 PMC7297507

[6] Sollars V, Lu X, Xiao L, Wang X, Garfinkel MD, Ruden DM. Evidence for an epigenetic mechanism by which Hsp90 acts as a capacitor for morphological evolution. Nature Genetics. 2003 Jan;33(1):70–4. 12483213 10.1038/ng1067

[7] Martínez-Campa C, Álvarez-García V, Alonso-González C, González A, Cos S. Melatonin and Its Role in the Epithelial-to-Mesenchymal Transition (EMT) in Cancer. Cancers. 2024 Feb 27;16(5):956. 38473317 10.3390/cancers16050956 PMC10931174

[8] Den Hollander P, Maddela JJ, Mani SA. Spatial and Temporal Relationship between Epithelial–Mesenchymal Transition (EMT) and Stem Cells in Cancer. Clinical Chemistry. 2024 Jan;70(1):190–205. 38175600 10.1093/clinchem/hvad197 PMC11246550

[9] Sumi MP, Ghosh A. Hsp90 in human diseases: Molecular mechanisms to therapeutic approaches. Cells. 2022 Mar 12;11(6):976. 35326427 10.3390/cells11060976 PMC8946885

[10] Fu X, Liu J, Yan X, DiSanto ME, Zhang X. Heat shock protein 70 and 90 family in prostate cancer. Life. 2022 Sep 26;12(10):1489. 36294924 10.3390/life12101489 PMC9605364

[11] Seclì L, Fusella F, Avalle L, Brancaccio M. The dark-side of the outside: How extracellular heat shock proteins promote cancer. Cellular and Molecular Life Sciences. 2021 May;78(9):4069–83. 33544155 10.1007/s00018-021-03764-3 PMC8164615

[12] Tian Y, Wang C, Chen S, Liu J, Fu Y, Luo Y. Extracellular Hsp90α and clusterin synergistically promote breast cancer epithelial-to-mesenchymal transition and metastasis via LRP1. Journal of Cell Science. 2019 Aug 1;132(15):jcs228213. 31273033 10.1242/jcs.228213

[13] Liu K, Chen J, Yang F, Zhou Z, Liu Y, Guo Y, BJ-B11, an Hsp90 inhibitor, constrains the proliferation and invasion of breast cancer cells. Frontiers in Oncology. 2019 Dec 18;9:1447. 31921692 10.3389/fonc.2019.01447 PMC6930179

[14] Chong KY, Kang M, Garofalo F, Ueno D, Liang H, Cady S, Inhibition of heat shock protein 90 suppresses TWIST1 transcription. Molecular Pharmacology. 2019 Aug 1;96(2):168–79. 31175180 10.1124/mol.119.116137

[15] Zhang A, Qi X, Du F, Zhang G, Li D, Li J. PNSA, a novel C-terminal inhibitor of HSP90, reverses epithelial–mesenchymal transition and suppresses metastasis of breast cancer cells in vitro. Marine Drugs. 2021 Feb 20;19(2):117. 33672529 10.3390/md19020117 PMC7923764

[16] Wu Q, Yang Z, Nie Y, Shi Y, Fan D. Multi-drug resistance in cancer chemotherapeutics: mechanisms and lab approaches. Cancer Letters. 2014 Jun 1;347(2):159–66. 24657660 10.1016/j.canlet.2014.03.013

[17] Bukowski K, Kciuk M, Kontek R. Mechanisms of multidrug resistance in cancer chemotherapy. International Journal of Molecular Sciences. 2020 May 2;21(9):3233. 32370233 10.3390/ijms21093233 PMC7247559

[18] You D, Richardson JR, Aleksunes LM. Epigenetic regulation of multidrug resistance protein 1 and breast cancer resistance protein transporters by histone deacetylase inhibition. Drug Metabolism and Disposition. 2020 Jun 1;48(6):459–80. 32193359 10.1124/dmd.119.089953 PMC7250367

[19] Ehrlich M DNA hypermethylation in disease: mechanisms and clinical relevance. Epigenetics. 2019 Dec 2;14(12):1141–63. 31284823 10.1080/15592294.2019.1638701 PMC6791695

[20] Lawag AA, Napper JM, Hunter CA, Bacon NA, Deskins S, El-Hamdani M, HSP90 inhibition and cellular stress elicits phenotypic plasticity in hematopoietic differentiation. Cellular Reprogramming. 2017 Oct 1;19(5):311–23. 28910138 10.1089/cell.2017.0001 PMC5650721

[21] Zabinsky RA, Mason GA, Queitsch C, Jarosz DF. It’s not magic–Hsp90 and its effects on genetic and epigenetic variation. Seminars in Cell & Developmental Biology. 2019 Apr 1;88:21–35. 29807130 10.1016/j.semcdb.2018.05.015 PMC6281791

[22] Birbo B, Madu EE, Madu CO, Jain A, Lu Y. Role of HSP90 in Cancer. International Journal of Molecular Sciences. 2021 Sep 25;22(19):10317. 34638658 10.3390/ijms221910317 PMC8508648

[23] Yin L, Yang Y, Zhu W, Xian Y, Han Z, Huang H, Heat Shock Protein 90 Triggers Multi-Drug Resistance of Ovarian Cancer via AKT/GSK3β/β-Catenin Signaling. Frontiers in Oncology. 2021 Mar 2;11:620907. 33738259 10.3389/fonc.2021.620907 PMC7960917

[24] Calderwood SK, Khaleque MA, Sawyer DB, Ciocca DR. Heat shock proteins in cancer: chaperones of tumorigenesis. Trends in Biochemical Sciences. 2006 Mar 1;31(3):164–72. 16483782 10.1016/j.tibs.2006.01.006

[25] Audia JE, Campbell RM. Histone modifications and cancer. Cold Spring Harbor Perspectives in Biology. 2016 Apr 1;8(4):a019521. 27037415 10.1101/cshperspect.a019521 PMC4817802

[26] Krämer OH, Mahboobi S, Sellmer A. Drugging the HDAC6–HSP90 interplay in malignant cells. Trends in Pharmacological Sciences. 2014 Oct 1;35(10):501–9. 25234862 10.1016/j.tips.2014.08.001

[27] Li QQ, Hao JJ, Zhang Z, Krane LS, Hammerich KH, Sanford T, Proteomic analysis of proteome and histone post-translational modifications in heat shock protein 90 inhibition-mediated bladder cancer therapeutics. Scientific Reports. 2017 Mar 15;7(1):201. 28298630 10.1038/s41598-017-00143-6 PMC5427839

[28] Dai C, Sampson SB. HSF1: guardian of proteostasis in cancer. Trends in Cell Biology. 2016 Jan 1;26(1):17–28. 26597576 10.1016/j.tcb.2015.10.011 PMC4722819

[29] Romero R, Sayin VI, Davidson SM, Bauer MR, Singh SX, LeBoeuf SE, Keap1 loss promotes Kras-driven lung cancer and results in dependence on glutaminolysis. Nature Medicine. 2017 Nov 1;23(11):1362–8. 10.1038/nm.4407 PMC5677540 28967920

[30] Jaeger AM, Whitesell L. HSP90: enabler of cancer adaptation. Annual Review of Cancer Biology. 2019 Mar 4;3:275–97.

[31] Li L, Wang L, You QD, Xu XL. Heat shock protein 90 inhibitors: an update on achievements, challenges, and future directions. Journal of Medicinal Chemistry. 2019 Oct 30;63(5):1798–822. 31663736 10.1021/acs.jmedchem.9b00940

[32] Rutherford SL, Lindquist S. Hsp90 as a capacitor for morphological evolution. Nature. 1998 Nov 26;396(6709):336–42. 9845070 10.1038/24550

[33] Queitsch C, Sangster TA, Lindquist S. Hsp90 as a capacitor of phenotypic variation. Nature. 2002 Jun 6;417(6889):618–24. 12050657 10.1038/nature749

[34] Whitesell L, Santagata S, Mendillo ML, Lin NU, Proia DA, Lindquist S. HSP90 empowers evolution of resistance to hormonal therapy in human breast cancer models. Proceedings of the National Academy of Sciences. 2014 Dec 23;111(51):18297–302. 10.1073/pnas.1421323111 PMC4280614 25489079

[35] Hanahan D, Weinberg RA. The hallmarks of cancer. Cell. 2000 Jan 7;100(1):57–70. 10647931 10.1016/s0092-8674(00)81683-9

[36] Hanahan D, Weinberg RA. Hallmarks of cancer: the next generation. Cell. 2011 Mar 4;144(5):646–74. 21376230 10.1016/j.cell.2011.02.013

[37] Kamal A, Thao L, Sensintaffar J, Zhang L, Boehm MF, Fritz LC, A high-affinity conformation of Hsp90 confers tumour selectivity on Hsp90 inhibitors. Nature. 2003 Sep 25;425(6956):407–10. 14508491 10.1038/nature01913

[38] Herbst RS, Morgensztern D, Boshoff C. The biology and management of non-small cell lung cancer. Nature. 2018 Jan 25;553(7689):446–54. 29364287 10.1038/nature25183

[39] Volm M, Mattern J, Samsel B. Overexpression of P-glycoprotein and glutathione S-transferase-π in resistant non-small cell lung carcinomas of smokers. British Journal of Cancer. 1991 Oct;64(4):700–4. 1680367 10.1038/bjc.1991.384 PMC1977695

[40] Oshika Y, Nakamura M, Tokunaga T, Fukushima Y, Abe Y, Ozeki Y, Multidrug resistance-associated protein and mutant p53 protein expression in non-small cell lung cancer. Modern pathology: an official journal of the United States and Canadian Academy of Pathology, Inc. 1998 Nov 1;11(11):1059–63. 9831202

[41] Ota E, Abe Y, Oshika Y, Ozeki Y, Iwasaki M, Inoue H, Expression of the multidrug resistance-associated protein (MRP) gene in non-small-cell lung cancer. British Journal of Cancer. 1995 Sep;72(3):550–4. 7669560 10.1038/bjc.1995.372 PMC2033872

[42] Berger W, Setinek U, Hollaus P, Zidek T, Steiner E, Elbling L, Multidrug resistance markers P-glycoprotein, multidrug resistance protein 1, and lung resistance protein in non-small cell lung cancer: prognostic implications. Journal of Cancer Research and Clinical Oncology. 2005 Jun;131:355–63. 15856298 10.1007/s00432-004-0653-9 PMC12161247

[43] Sosa Iglesias V, Giuranno L, Dubois LJ, Theys J, Vooijs M. Drug resistance in non-small cell lung cancer: a potential for NOTCH targeting?. Frontiers in Oncology. 2018 Jul 24;8:267. 30087852 10.3389/fonc.2018.00267 PMC6066509

[44] Shanker M, Willcutts D, Roth JA, Ramesh R. Drug resistance in lung cancer. Lung Cancer: Targets and Therapy. 2010 May 8:23–36. PMC5312467 28210104

[45] He J, Fortunati E, Liu DX, Li Y. Pleiotropic roles of ABC transporters in breast cancer. International Journal of Molecular Sciences. 2021 Mar 21;22(6):3199. 33801148 10.3390/ijms22063199 PMC8004140

[46] American Cancer Society Breast Cancer Survival Rates. [cited 2024 April 9]; Available from: https://www.cancer.org/cancer/types/breast-cancer/understanding-a-breast-cancer-diagnosis/breast-cancer-survival-rates.html.

[47] Kajiyama H, Shibata K, Terauchi M, Yamashita M, Ino K, Nawa A, Chemoresistance to paclitaxel induces epithelial-mesenchymal transition and enhances metastatic potential for epithelial ovarian carcinoma cells. International Journal of Oncology. 2007 Aug 1;31(2):277–83. 17611683

[48] Karagiannis GS, Pastoriza JM, Wang Y, Harney AS, Entenberg D, Pignatelli J, Neoadjuvant chemotherapy induces breast cancer metastasis through a TMEM-mediated mechanism. Science Translational Medicine. 2017 Jul 5;9(397):eaan0026. 28679654 10.1126/scitranslmed.aan0026 PMC5592784

[49] Volk-Draper L, Hall K, Griggs C, Rajput S, Kohio P, DeNardo D, Paclitaxel therapy promotes breast cancer metastasis in a TLR4-dependent manner. Cancer Research. 2014 Oct 1;74(19):5421–34. 25274031 10.1158/0008-5472.CAN-14-0067 PMC4185415

[50] Garraway LA, Jänne PA. Circumventing cancer drug resistance in the era of personalized medicine. Cancer Discovery. 2012 Mar 1;2(3):214–26. 22585993 10.1158/2159-8290.CD-12-0012

